# Integration of nuclear Ca^2+^ transients and subnuclear protein shuttling provides a novel mechanism for the regulation of CREB-dependent gene expression

**DOI:** 10.1007/s00018-023-04876-8

**Published:** 2023-07-25

**Authors:** Anna Karpova, Sebastian Samer, Rabia Turacak, PingAn Yuanxiang, Michael R. Kreutz

**Affiliations:** 1 RG Neuroplasticity, Leibniz Institute for Neurobiology, 39118 Magdeburg, Germany; 2 Center for Behavioral Brain Sciences, Otto von Guericke University, 39106 Magdeburg, Germany; 3 Leibniz Group ‘Dendritic Organelles and Synaptic Function’, Center for Molecular Neurobiology, ZMNH, University Medical Center Hamburg-Eppendorf, 20251 Hamburg, Germany

**Keywords:** Jacob, LaminB1, Nuclear calcium transients, NMDAR, CREB

## Abstract

Nuclear Ca^2+^ waves elicited by NMDAR and L-type voltage-gated Ca^2+^-channels as well as protein transport from synapse-to-nucleus are both instrumental in control of plasticity-related gene expression. At present it is not known whether fast [Ca^2+^]_n_ transients converge in the nucleus with signaling of synapto-nuclear protein messenger. Jacob is a protein that translocate a signalosome from N-methyl-D-aspartate receptors (NMDAR) to the nucleus and that docks this signalosome to the transcription factor CREB. Here we show that the residing time of Jacob in the nucleoplasm strictly correlates with nuclear [Ca^2+^]_n_ transients elicited by neuronal activity. A steep increase in [Ca^2+^]_n_ induces instantaneous uncoupling of Jacob from LaminB1 at the nuclear lamina and promotes the association with the transcription factor cAMP-responsive element-binding protein (CREB) in hippocampal neurons. The size of the Jacob pool at the nuclear lamina is controlled by previous activity-dependent nuclear import, and thereby captures the previous history of NMDAR-induced nucleocytoplasmic shuttling. Moreover, the localization of Jacob at the nuclear lamina strongly correlates with synaptic activity and [Ca^2+^]_n_ waves reflecting ongoing neuronal activity. In consequence, the resulting extension of the nuclear residing time of Jacob amplifies the capacity of the Jacob signalosome to regulate CREB-dependent gene expression and will, thereby, compensate for the relatively small number of molecules reaching the nucleus from individual synapses.

**Supplementary Information:**

The online version contains supplementary material available at 10.1007/s00018-023-04876-8.

## Introduction

De novo transcription of DNA is a fundamental requirement for the formation of long-term memory, where it is instrumental in memory consolidation [1]. Local signaling events at synapses must be communicated to the nucleus to elicit transcriptional responses. A central component of these signaling events is the activation of NMDAR that triggers different excitation-transcription pathways, which are eventually integrated in the nucleus to produce an adequate genomic response. Part of fast electrochemical signal propagation to the soma is opening of L-type voltage-gated Ca^2+^ channels, that is instrumental for backpropagating action potentials and dendritic Ca^2+^ spikes [2, 3]. Somatic Ca^2+^ can either enter the nucleus or trigger the entry of transcription factors from the soma [4–6]. Moreover, the nuclear envelope is a Ca^2+^ store, that serves as a supplier of nuclear Ca^2+^ [7, 8]. Increased nuclear Ca^2+^ transients elicited by neuronal activity are crucial for the regulation of gene expression [4, 9–12]. In fact, in hippocampal neurons, nuclear calcium is one of the most potent signals regulating gene expression that control adaptive responses to synaptic activity, cell survival, and cell death [5, 12–23].

Taken together, these events constitute the fast electrochemical signaling, which alone is insufficient to cause a full transcriptional response to synaptic activity [24, 25]. Long distance transport of macromolecular protein complexes provides a second means of signaling on a delayed time scale that does not only sustain the transcriptional response, but further direct it, as synapto-nuclear protein messengers can encode and convey more precise instructions [26–29]. Thus, such proteins allow for local encoding of signals at the site of origin and decoding in the nucleus. In the past decade, several studies including work from our lab have proposed mechanisms of activity-dependent transport of synaptic proteins to the nucleus [26, 30–36]. We could show that proteins that directly interact with NMDAR subunits can be transported to the nucleus. Following nuclear import, these proteins associate with transcription factor complexes and can induce sustained changes in gene expression [30, 34, 35].

Jacob is one of those synapto-nuclear protein messengers that transduce signals from GluN2B-containing NMDAR to CREB-dependent gene expression [30, 31, 37]. Depending upon the synaptic or extrasynaptic origin of those signals, Jacob docks a signalosome containing different protein components to CREB that either promote expression of plasticity-related genes or result in CREB shut-off, the transcriptional inactivation of CREB [30, 31, 37]. Interestingly, following nuclear import, Jacob undergoes a stepwise subnuclear redistribution [38]. It first associates with the nuclear lamina (NL), a meshwork of intermediate filament-like proteins called Lamins that are tightly embedded into the inner nuclear membrane (INM). Lamins function as scaffold for scavenging of signaling molecules involved in gene regulation and their silencing through the association with heterochromatin [39–41]. Nuclear Lamins contribute to the organization of chromatin into lamina-associated domains (LADs), representing the interface between chromatin and the inner part of the nuclear envelope (NE) [42–45].

Here we show that the dissociation of Jacob from the NL strongly correlates with nuclear [Ca^2+^]_n_ transients. Proximity ligation assays revealed a Ca^2+^-dependent dissociation of Jacob from LaminB1 which is accompanied by an increased association of Jacob with the transcription factor CREB. Intriguingly, the pattern of nuclear [Ca^2+^]_n_ transients is strictly correlated with Jacob’s subnuclear distribution. When we induced long-term potentiation with field stimulation in hippocampal primary neurons, we observed an immediate dissociation of Jacob from NL and a subsequent accumulation with the nuclear matrix. Moreover, the dissociation of Jacob from the NL strictly follows the pattern of nuclear [Ca^2+^]_n_ elicited by different types of synaptic stimulation. We speculate that the Ca^2+^-dependent association with LaminB1 controls the nuclear function of Jacob inasmuch as its stimulus-dependent availability in the nuclear matrix will contribute to a specific pattern of CREB-dependent gene regulation reflecting different patterns of nuclear [Ca^2+^]_n_ transients.

## Materials and methods

### Neuronal and HEK293T cell culture and transfection

Rat hippocampal primary cultures were prepared from E18-E19 embryos as described previously [31]. Wistar rats were bred and housed in the animal facility of the Leibniz Institute for Neurobiology, Magdeburg. Animal handling and experiments were conducted following ethical animal research standards defined by German Law. Neurons were transfected with Jacob-GFP or Jacob-CyRFP2 constructs at DIV15/17 using lipofectamine2000 (Invitrogen) according to published work [46]. HEK293T cell culture and transfection were described before [38]. For the synaptic activity reporter assay, hippocampal neurons were co-transfected with 1,8 µg/well plasmid DNA of Jacob-GFP expressed under the CMV promoter and 1,8 µg /well of Synaptic Activity-Responsive Element reporter (SARE-ArcMin-Luc, [47]). Next day, neurons were fixed with 4% PFA and the expression of the reporter was estimated by immunodetection of luciferase.

### Confocal laser scanning FRAP imaging

FRAP experiments were performed using a FRAP module installed at the Leica TCS SP5-STED confocal system (Leica-Microsystems, Mannheim, Germany) equipped with an Argon laser (488). Neurons were transfected at DIV16, one day before the imaging experiment, with a plasmid expressing full length Jacob fused to GFP and FRAP was performed with a 63 × objective in conditioned medium at 37 °C and 5% CO_2_. Images were acquired in one focal plane with 5 frames before bleaching at 1 Hz, 9 frames during bleaching at minimized frequency, and 60 frames at 1 Hz after bleaching. Two bar-shaped bleach zones denoted as regions of interest (ROIs) were made across the nuclear envelope of Jacob-GFP expressing neurons with an intense (set to 100%.) argon laser beam (488 nm). Fluorescence recovery within each ROI was measured over time, normalized to overall sample bleaching, and the averaged calculation of the recovery was plotted.

FRAP was calculated following an equation with ${I}_{\mathrm{FRAP}(T)}$ as the corrected fluorescence intensity recovery in the ROI, ${I}_{\mathrm{FRAP}(\mathrm{pre})}$ as the fluorescence intensity before bleaching in the ROI, ${I}_{\mathrm{REF}(\mathrm{pre})}$ as the fluorescence intensity before bleaching in a nearby non-bleached reference area on the same cell, ${I}_{\mathrm{FRAP}(t)}$ as the fluorescence intensity at a given timepoint in the ROI, ${I}_{\mathrm{BCG}(t)}$ as the background signal at a given timepoint in an area outside the neuron, and ${I}_{\mathrm{REF}(t)}$ as the fluorescence intensity at a given timepoint in the non-bleached reference area on the same cell [48].

$${I}_{\mathrm{FRAP}(T)}=\frac{{I}_{\mathrm{REF}(\mathrm{pre})}}{{I}_{\mathrm{REF}(t)}-{I}_{\mathrm{BCG}(t)}}*\frac{{I}_{\mathrm{FRAP}\left(t\right)}-{I}_{\mathrm{BCG}\left(t\right)}}{{I}_{\mathrm{FRAP}\left(\mathrm{pre}\right)}}.$$

### Confocal time-lapse imaging and field stimulation

Time-lapse imaging was performed in a single focal nuclear plane with ΔF spacing equal either 1 Hz or 2 Hz employing confocal microscopy** (**Leica TCS SP5-STED confocal system). To avoid fast bleaching, a low laser power illumination and Leica hybrid super-sensitivity detectors (HyD) for image acquisition were used enabling us to examine Jacob`s nuclear redistribution pattern at a low expression level. Jacob-GFP intensities at the nuclear envelope and within the nuclear matrix were measured as mean grey value in arbitrary units of pixel intensity using FIJI software [49] (https://fiji.sc) and normalized either to a single time point or to the averaged baseline.

Fluo-4 (AM) cell loading (1 μM) was performed 10 min before the image acquisition, subsequently washed out with conditional neurobasal media (Gibco). For the simultaneous detection of nuclear [Ca^2+^]_n_ dynamic and Jacob protein subnuclear redistribution, Fluo-4 (AM) and CyRFP2 were illuminated at the same time with Argon laser (488) and detected within the emission ranges, 520–550 nm and 570–615 nm accordingly.

Field stimulation was performed as described previously [34]. Briefly, a quick change 18 mm Chamber with Field Stimulation (RC-49MFS, Warner Instruments) was used for mounting coverslips with living neurons expressing Jacob-GFP or Jacob-CyRFP2. Master-8/U 8-channel programmable pulse generator (Science Products) and the constant-current stimulus isolation unit (SIU-102, Warner Instruments) were used as described before [34]. Long-term potentiation (LTP) was induced by applying current pulses 18 s@50 Hz via a field stimulation electrode [50].

### Acute hippocampal slice preparation and stimulation protocols

Hippocampi of 21–23 days old rats were dissected from the brain and acute 350 μm thick slices were prepared using a McILWAIN tissue chopper (Mickle laboratory engineering Co. LTD., Gomshall, UK) in cold artificial cerebrospinal fluid (ACSF, 4 °C) saturated with 95%O_2_–5%CO_2_ containing: 110 mM NaCl, 2.5 mM KCl, 1.5 mM MgSO_4_·2H_2_O, 2.5 mM CaCl_2_, 1.24 mM KH_2_PO_4_, 27.4 mM NaHCO_3_, and 10 mM D-glucose (pH 7.3) [51]. Prior to stimulation, hippocampal slices were incubated for 90 min in carbogenated artificial cerebrospinal fluid (ACSF, containing in mM: 110 NaCl, 5 KCl, 2.5 CaCl_2_·2H_2_O, 1.5 MgSO_4_·7H_2_O, 1.24 KH_2_PO_4_, 10 glucose, 27.4 NaHCO_3_, pH 7.3) at 31 ± 1 °C on submerged grids floating in the 15 ml glass beaker. Following incubation, we treated slices with bath NMDA (100 μM) for 5 min to secure an increase in nuclear Ca^2+^ transients in most CA1 pyramidal neurons in a plateau-like fashion to estimate the Ca^2+^ dependent redistribution of Jacob. After fixation, slices were processed for proximity ligation assays in combination with immunohistochemistry.

Field excitatory postsynaptic potential (fEPSP) was recorded as described previously [52]. Following a stable baseline period of 15 min, LTP was induced in the Stratum Radiatum by electrical theta burst stimulation (TBS) of Schaffer-collateral fibers consisting of four trains of theta bursts. Each train comprised of 10 bursts at 5 Hz (5 stimuli per burst) was given at 100 Hz and repeated every 20 s. Three minutes after TBS, slices were fixed in 4% PFA in 1 × PBS overnight and transferred into 30% sucrose in PBS for 24 h. After that, slices were cut frozen on a cryostat (Leica CM3050S, Leica Biosystems) at 40 μm sections and proceeded for proximity ligation between endogenous Jacob and LaminB1, endogenous Jacob and CREB, as well as pS180-Jacob and CREB.

### Antibodies, immunocytochemistry, immunohistochemistry

Custom-made, affinity-purified rabbit polyclonal antibodies generated against two short amino acid stretches of rat Jacob (285–299 and 400–314, Jb150) and against the phosphorylated peptide LVPGPSpPRAFG corresponding to amino acids 178–187 of the rat Jacob sequence (ProteoGenix) have been described previously [31, 37, 38, 52, 53]. The following commercial primary antibodies were used in the current study: mouse monoclonal anti-LaminB1 (ProteinTeck, 3C10G12; #66095-1-Ig; RRID: AB_2721256); mouse monoclonal anti-CREB (ThermoFisher Scientific, LB9, #MA1-083; RRID: AB_558523); mouse monoclonal anti-Luciferase (Calbiochem, #OB09; RRID: AB_564792); monoclonal anti-MAP2, Alexa Fluor 488 conjugated (Merck, #MAB 3418X) and rabbit polyclonal anti-MAP2 (Sigma-Aldrich, #AB5622-I; RRID: AB_2800501). 4',6-Diamidino-2-phenylindol (DAPI) and DRAQ5^™^ were used as a nuclear counterstain.

For immunocytochemical analysis, neurons were fixed with 4% paraformaldehyde (PFA) for 5 min, washed in PBS, and proceeded with a heat-based antigen retrieval. This included the treatment of neurons with 10 μM sodium citrate solution (pH 9) for 30 min at 80 °C. The pH of the sodium citrate (Fluka) solution was adjusted with 5 M NaOH. Next, neurons were permeabilized with 0.1% Triton X100 in PBS for 10 min, washed and incubated for at least 45 min in a blocking buffer (2% glycine, 0.2% gelatin, 2% BSA, and 50 mM NH_3_Cl (pH 7.4)). Primary antibody was diluted in the blocking buffer (60 µl/coverslip) and incubated overnight at 4 °C. DAPI was applied for 10 min at a dilution of 1:1000 in PBS. Coverslips were mounted with Mowiol 4–88 (Calbiochem/Merck Chemicals Ltd., Nottingham United Kingdom).

### Proximity ligation assay

For PLA, neurons and 40 μm hippocampal sections were treated as described above including a heat-based antigen retrieval with 10 μM sodium citrate solution (pH 9) for 30 min at 80 °C. After incubation with primary antibodies 24 to 72 h at 4 °C, coverslips and sections were washed with Duolink wash buffer A (Sigma-Aldrich) and incubated with Duolink PLA probes PLUS and MINUS in a humidity chamber for 1 h at 37 °C. For ligation of the probes, coverslips and slices were washed with buffer A and then incubated with ligase for 1 h at 37 °C. For amplification step, coverslips and slices were incubated with polymerase and Duolink FarRed Detection Reagent for 100 min at 37 °C. Prior to incubation with other antibodies for immunocytochemistry and immunohistochemistry as well as DAPI counterstaining, coverslips and slices were washed with PBS and then mounted with Mowiol 4–88. Averaged intensity of PLA signal was quantified and normalized to untreated controls.

### Compounds and pharmacological treatments

The Ca^2+^ ionophore ionomycin (1 μM) was obtained from Invitrogen (#I24222). The cell-permeable far-red fluorescent DNA dye 1,5-bis[13]-4,8-dihydroxyanthracene-9,10-dione (DRAQ5^™^) was obtained from abcam (ab108410). 4-Aminopyridine (4-AP, 2.5 mM) was obtained from Sigma (Munich, Germany). NMDA (50 μM, 100 μM), bicuculline methiodide (50 μM), Ifenprodil (5 μM) were purchased from Tocris.

### Statistical analysis

Blind data collection was employed as a protection against bias. Statistical analyses were performed with the paired Student’s *t* test, nested *t* test using GraphPad Prism 8 (GraphPad Software). Error bars indicate SEM and sample numbers are either indicated in the graph or in the figure legend. For correlation analysis between Fluo-4 (AM) and Jacob-CyRFP intensity changes, time-lapse recordings were corrected for bleaching and normalized to signal intensities between events. Jacob signal intensities in the nuclear matrix were plotted over Fluo-4 (AM) signal intensities, and a linear regression model and Pearson correlation coefficients were computed to detect correlation.

## Results

### The association of Jacob with the nuclear envelope is highly dynamic

We have shown previously that the nuclear import of Jacob critically relies on the activation of GluN2B-containing NMDAR receptors [34]. Moreover, we found that the size of the Jacob pool at the nuclear lamina is controlled by activity of GluN2B-, but not GluN2A-containing NMDAR [38]. Therefore, we reasoned that the subsequent sequestration of Jacob at the INM could potentially provide a read-out of a neuron’s recent history in terms of NMDAR activity [31, 37, 38, 52, 53]. Jacob associates to the NL via direct binding to LaminB1 [38, 54]. At present, it is unclear whether the association with the NL is dynamic and whether the accumulating pool at the NL might foster the regulation of gene expression.

**Fig. 1:**
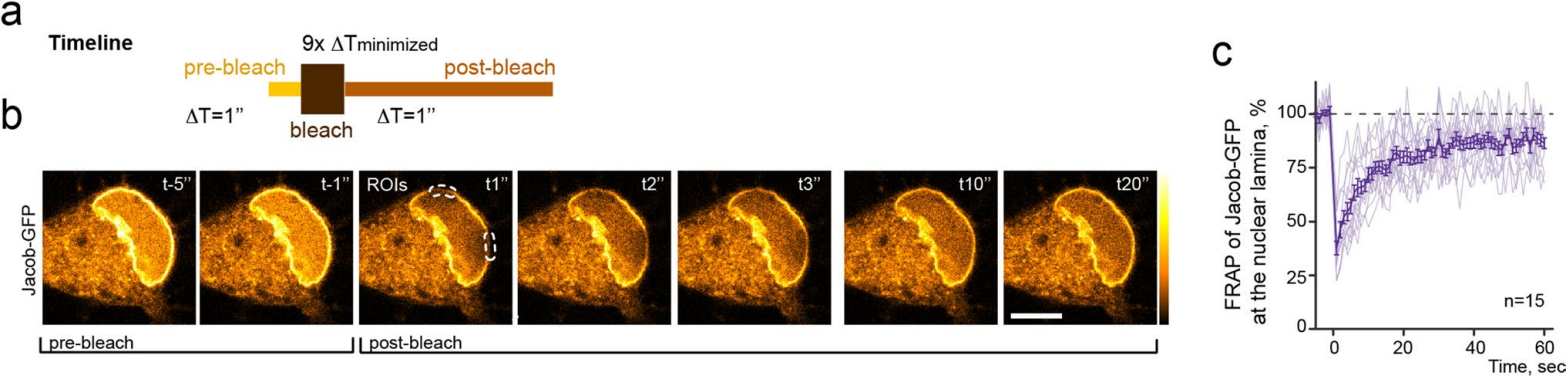
The association of Jacob with the nuclear envelope is dynamic. **a** The schematic represents the timeline of the FRAP experiment. **b** Representative confocal image sequence showing hippocampal neuron (DIV17) expressing Jacob-GFP enriched at the NL. Two pre-bleach images, the immediate post-bleach image (1 s) and multiple images taken during the recovery period, are shown. Dashed lines denote the photobleached regions of interest (ROIs). Scale bar = 10 μm. **c** Fluorescence recovery kinetics of Jacob-GFP at the NL in neurons (*n* = 15). Fluorescence intensities were background corrected, normalized, and plotted versus time (seconds). The dark violet plot represents the mean values of recovery (± SEM); light violet lines represent individual FRAP measures

To address these questions, we first expressed the protein in mature hippocampal neurons, photobleached a small area within the nuclear envelope and plotted the fluorescence recovery after photobleaching (FRAP; Fig. 1a–c; Suppl. Movie 1). LaminB1 is tightly anchored to the INM and exhibits very low if any lateral diffusion [55]. Nonetheless, Jacob-GFP fluorescence within the nuclear rim shows a stunningly quick recovery within a few seconds, indicating that no stable pool resides at the INM and that the exchange between “free” and “sequestered” Jacob at the NL occurs fast and might have regulatory impact for its nuclear function.

### Nucleoplasmic retention of Jacob matches the transient increase in nuclear [Ca^2+^]_n_

**Fig. 2:**
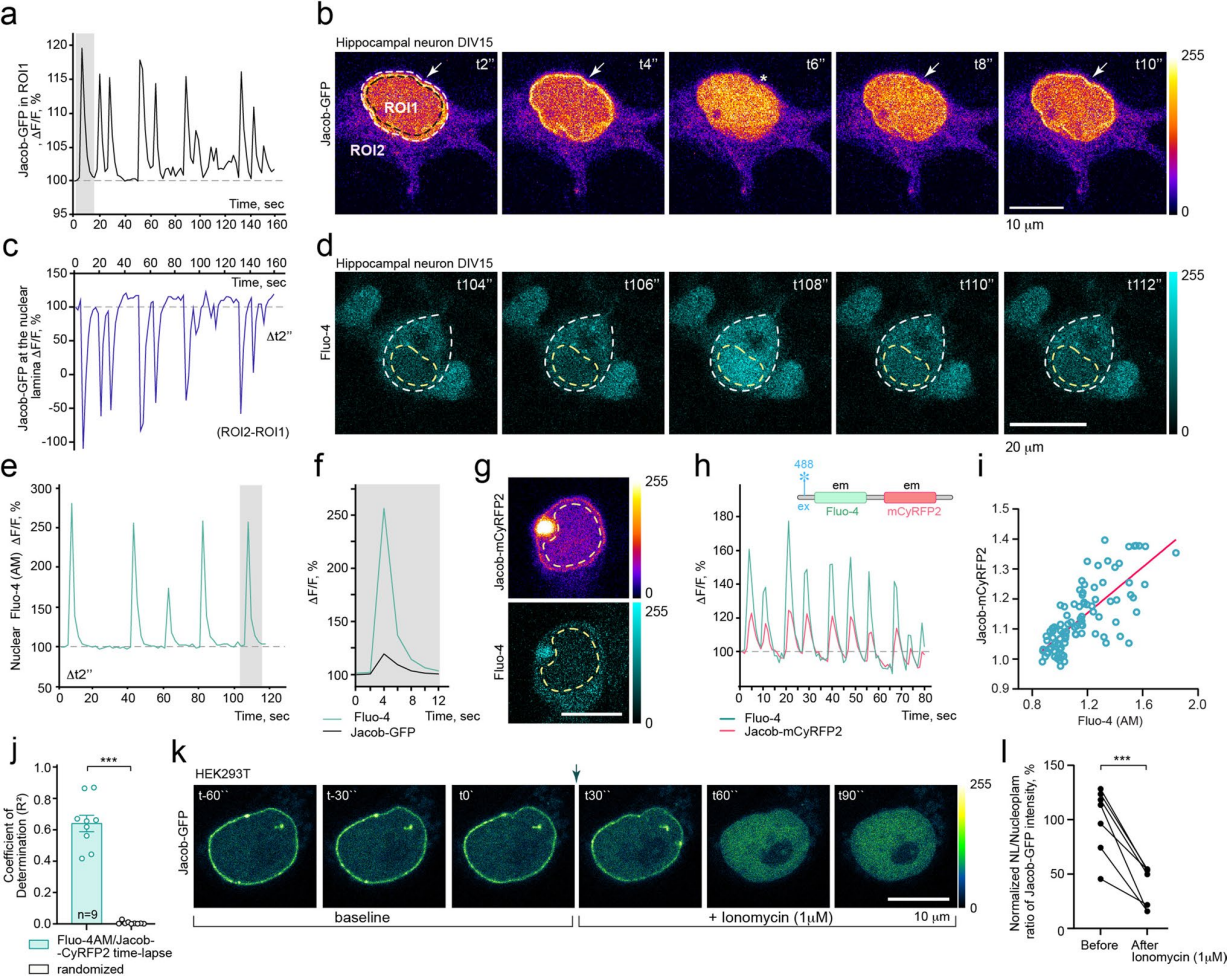
Dissociation of Jacob from the nuclear lamina and its retention in the nuclear matrix is regulated by [Ca^2+^]_n_. **a** Representative trace of Jacob-GFP fluorescence within the nuclear matrix of hippocampal neurons under basal glutamatergic transmission. **b** Depicted are confocal image frames corresponding to 8 s of recording (grey bar in **a**). The arrow points to the NL. ROI1 represents the region of interest excluding the nuclear rim; ROI2 includes the NL. Asterisk indicates the image with reduced Jacob-GFP intensity at the NL. Scale bar = 10 μm. **c** The panel represents fluorescence vs. time plot of fluorescence intensity at the NL. Note that decays in fluorescence intensity at the nuclear envelope correspond to rises in Jacob-GFP intensity within the nuclear matrix (**a**). **d** Depicted are confocal image frames of the hippocampal neuron filled with cell-permeable single wavelength Ca^2+^ indicator Fluo-4 (AM). The time points in the panel correspond to the grey bar in plot **e**. **e** Changes in fluorescent intensities due to rises of [Ca^2+^]_n_ as depicted with the Z-axis profile plot from hippocampal neurons filled with Ca^2+^ indicator Fluo-4 (AM). **f** A spontaneous intensity fluctuation of Jacob-GFP within the nuclear matrix (grey box, panel **a**) shows a similar temporal pattern with a spontaneous nuclear Ca^2+^ wave (grey box, panel **e**). Note that the amplitude of Jacob-GFP intensity is relative and largely depends on the protein expression level. Also, changes in the amplitude of Fluo-4 (AM) are not comparable between experiments and depends on dye loading (**f–h**). Schematic drawing in (**h**) represents simultaneous excitation and detection of Fluo-4 (AM) and Jacob-CyRFP2. **g–j** Simultaneous time-lapse image acquisition detecting correlation between rises in nuclear calcium and increased nuclear fluorescent intensity of Jacob-CyRFP2. Pearson *r* = 0.9324, *p* < 0.0001. **j** Coefficient of determination (*R*^2^) calculated for Fluo-4AM and Jacob-CyRFP2 in neuronal nuclei during time-lapse image acquisition. For control measurements, images were randomly distributed along the time axis (paired Student‘s *t* test; *n* = 9). **k–l** Time-lapse imaging showing subnuclear redistribution of Jacob-GFP in HEK293T cells after the treatment with effective Ca^2+^ ionophore ionomycin (1 μM). **l** The graph represents relative changes in NL/Nucleoplasm Jacob’s ratios before and after the treatment with ionomycin. ***p < 0.001 by paired *t* test

During the fast data acquisition in FRAP experiments, we noticed relatively rapid fluctuations in Jacob fluorescence intensity within the nuclear matrix and at the nuclear rim (Fig. 1c). When we evaluated the time course of nucleoplasmic retention with time-lapse imaging, we observed fluorescence intensity fluctuations of Jacob-GFP within the nucleoplasm that could last a couple of seconds. Moreover, these events appeared at different frequencies (Fig. 2a–b; Suppl. Movie 2; Suppl. Fig. 1a–b). The increase of Jacob-GFP fluorescence within the nucleoplasm was accompanied by a decrease in GFP fluorescence intensity at the INM, indicating a rapid redistribution of the protein within the nucleus (Fig. 2b–c; Suppl. Movie 2). We next reasoned that such rapid redistribution might closely resemble the dynamics and occurrence of nuclear Ca^2+^ transients ([Ca^2+^]_n_).

To test this hypothesis, in the next set of experiments, we loaded hippocampal neurons with the cell-permeable single wavelength Ca^2+^-indicator Fluo-4 (AM) and thereby detected local [Ca^2+^]_n_. Intriguingly, we found that the time course of Jacob`s redistribution to the nucleoplasm matched the time course of nuclear Ca^2+^ waves (Fig. 2d–f). To confirm the correlation between transient increases in nuclear [Ca^2+^]_n_ and nuclear residing time of Jacob in hippocampal neurons, we fused the monomeric cyan-excitable red fluorescent protein CyRFP2 to Jacob [56]. This approach allowed us to simultaneously determine protein redistribution and fluctuations in nuclear Ca^2+^ levels by Fluo-4 (AM). As expected, we observed a similar pattern of nuclear oscillations for the rise in [Ca^2+^]_n_ and changes in Jacob-CyRFP2 intensity within the nuclear matrix, suggesting a Ca^2+^-dependent regulation of Jacob`s subnuclear distribution (Fig. 2g–j).

The nuclear envelope is a Ca^2+^ store, that serves as a supplier of nuclear Ca^2+^ [7, 8]. In addition, the nuclear pore complex allows shuttling of [Ca^2+^] between the cytoplasm and nucleoplasm, and increased nuclear Ca^2+^ transients elicited by neuronal activity are crucial for the regulation of gene expression [4, 9–12]. Therefore, we hypothesized that a rise in nuclear [Ca^2+^]_n_ might trigger the dissociation of Jacob from the NL. We indeed found that Jacob associates with the nuclear envelope of HEK293T cells where the protein is not abundant if present at all [38] (Fig. 2k). As expected, treatment with the Ca^2**+**^ ionophore ionomycin (1 μM) boosted Jacob's intensity within the nuclear matrix and, concomitantly, reduced its intensity at the nuclear periphery (Fig. 2k–l).

### Dissociation of Jacob from LaminB1 and binding to CREB is regulated by [Ca^2+^]_n_ transients

The tail domain of LaminB1 is known to bind divalent cations, and [Ca^2+^] interferes with LaminB1 polymerization in vitro [54, 57], indicating that nuclear [Ca^2+^]_n_ will impact LaminB1 conformation. We, therefore, next addressed whether enhanced [Ca^2+^]_n_ transients are instrumental for the dissociation of Jacob from a preformed complex with LaminB1 and whether this dissociation is accompanied by an increased binding to the transcription factor CREB.

**Fig. 3:**
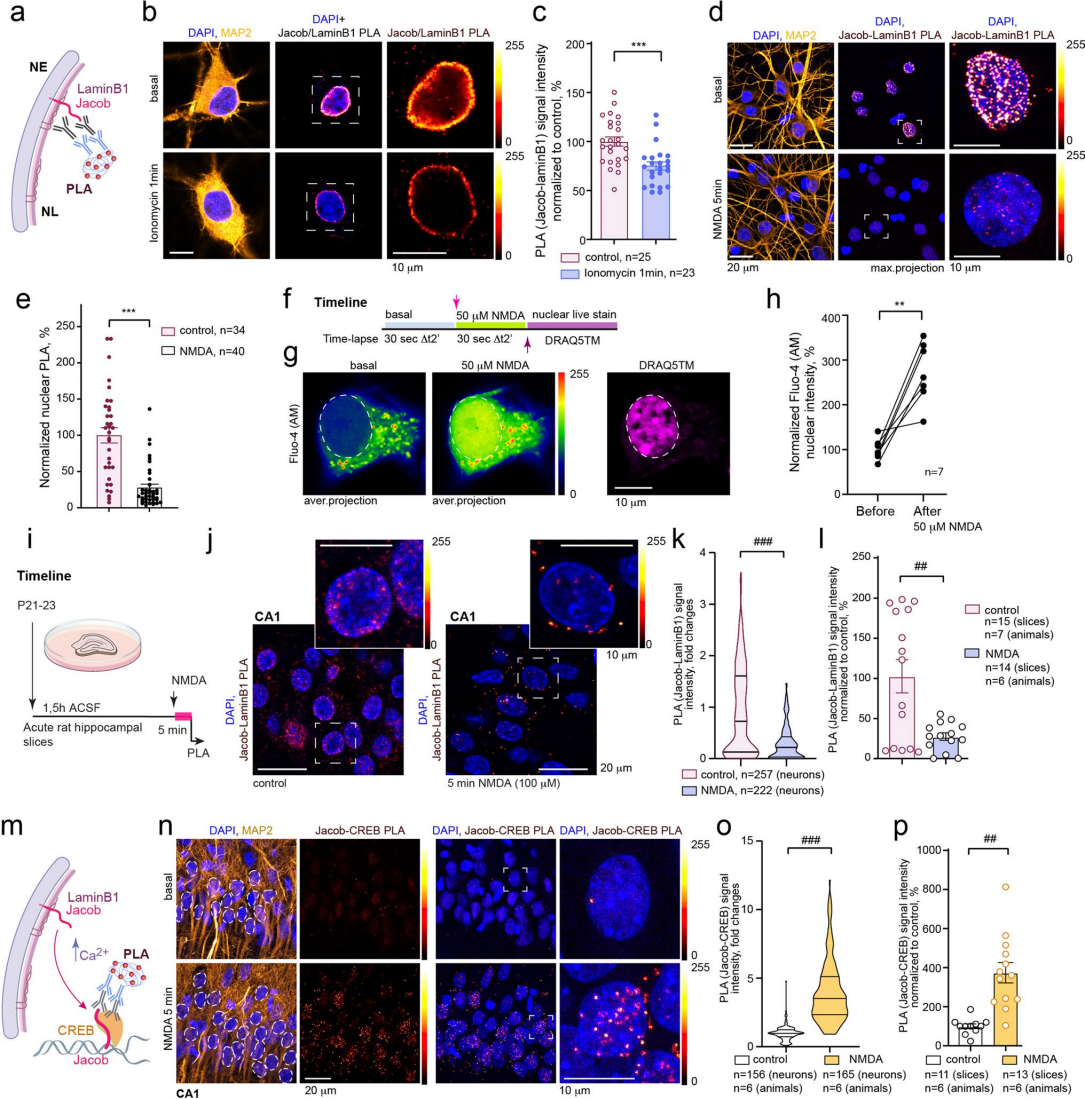
Increase in [Ca^2+^]_n_ results in dissociation of Jacob from LaminB1 and promotes binding of Jacob to CREB. **a** Schematic representations of Jacob–LaminB1 PLA assay. *NE*—nuclear envelope; *NL*—nuclear lamina. *PLA*—proximity ligation assay. **b**–**c** One minute treatment of hippocampal neurons with ionomycin (1 μM) results in the dissociation of Jacob from LaminB1, as revealed by substantial decreased PLA intensity at the nuclear envelope. Panel **b** depicts a single focal plane confocal image of cultured hippocampal neurons (DIV15) incubated with anti-Jacob (rb) and anti-LaminB1 (ms) antibodies and proceeded for proximity ligation followed by labeling with anti-MAP2 antibodies and DAPI; magnified image represents one focal plane through neuronal nuclei. Scale bars = 10 μm. **d**–**e** Short treatment of primary hippocampal neurons with bath NMDA (50 μM) results in decreased PLA intensity at the NL, indicating reduced association of Jacob with LaminB1. Depicted are representative confocal maximum intensity projection images of cultured hippocampal neurons detected with MAP2 staining. **f**–**h** The treatment of 15DIV hippocampal neurons with NMDA (50 μM) results in robust increase in nuclear [Ca^2+^]_n_. **f** The schematic represents the timeline of the experiment where time-lapse imaging was performed before and after NMDA (50 μM) treatment. DRAQ5^™^ DNA stain was added live for detection of nuclei. **g** Confocal averaged intensity projection images obtained from 30 s time-lapse recording. **h** The graph represents relative changes in Fluo-4 (AM) intensity before and after the treatment with 50 μM NMDA. **p < 0.01 by paired *t* test. **i**–**l** Jacob dissociates from LaminB1 in CA1 pyramidal neurons in acute hippocampal slices treated with bath NMDA as revealed by a significant reduction in PLA dots at the nuclear periphery. **k** ^###^*p* < 0.001, Nested *t* test **l** ^##^*p* < 0.01 Nested *t* test **m**–**p** Short treatment of acute hippocampal slices with bath NMDA dramatically increases association of Jacob with CREB in CA1 neurons. **o** ^###^*p* < 0.001, Nested *t* test **p** ^##^*p* < 0.01, Nested *t* test

Proximity ligation assays revealed a significant reduction in the association between endogenous LaminB1 and Jacob at the nuclear periphery already 1 min after the treatment of neurons with the calcium ionophore ionomycin (1 μM) (Fig. 3a–c). Treatment of neurons for 5 min with 50 μM NMDA was accompanied by a pronounced reduction of the Jacob–LaminB1 PLA signal at the nuclear envelope, indicating a dissociation of Jacob from LaminB1 upon stimulation (Fig. 3d–e + f–h).

We further substantiated these findings in acute hippocampal slices, a physiologically more relevant experimental system with preserved synaptic circuitry. Here we found that in CA1 neurons, endogenous Jacob dissociates from the complex with LaminB1 after brief bath application of bath NMDA (Fig. 3i–l). We have shown previously that Jacob transduces to the nucleus the origin of synaptic and extrasynaptic NMDAR signals [30, 31, 37]. In both cases, Jacob binds CREB, though it either stimulates or represses its transcriptional activity [30, 31]. Accordingly, we found that the dissociation of Jacob from the nuclear lamina after a short treatment with NMDA precedes an enhanced association with CREB in CA1 pyramidal neurons, as evidenced by Jacob-CREB proximity ligation with endogenous proteins (Fig. 3m–p). Collectively, these data suggest that the NL serves as a docking site for Jacob's intermediate sequestration. An increase in [Ca^2+^]_n_ transients liberates Jacob from the association with LaminB1, thereby promoting its binding to CREB.

### The pattern of synaptic stimulation resembles the pattern of Jacob’s dissociation from the nuclear lamina

**Fig. 4:**
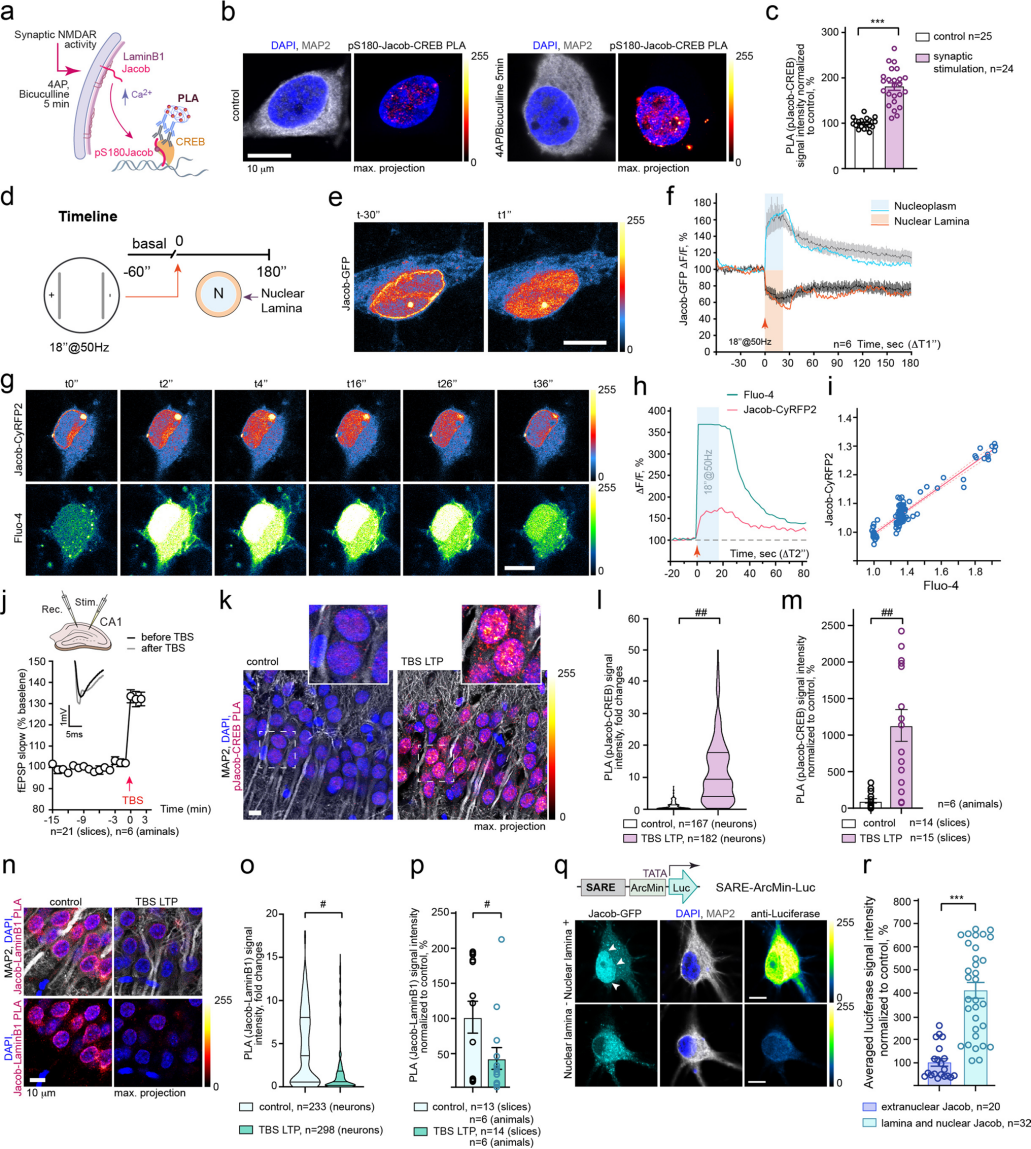
Field stimulation results in subnuclear redistribution of Jacob that correlates with enhanced [Ca^2+^]_n_. **a** Schematic representation of pS180-Jacob-CREB PLA assay. **b**–**c** Five-minute treatment of hippocampal neurons with 4AP/bicuculline (2,5 mM/ 50 μM) results in dramatic association of pS180-Jacob with CREB, as revealed by significant increase in PLA intensity within the nucleus. Panel **b** depicts maximum projection confocal image of cultured hippocampal neurons (DIV16) incubated with anti-pJacob (rb) and anti-CREB (ms) antibodies and proceeded for proximity ligation followed by labeling with anti-MAP2 antibodies and DAPI; Scale bar = 10 μm, ***p < 0.001 by *t* test. **d** Timeline of the experiment. **e** Confocal time-lapse images of hippocampal neuron overexpressing Jacob-GFP before and after field stimulation (18’’@50 Hz) showed an increase in fluorescent intensity at the nuclear matrix and a decrease in intensity at the nuclear envelope upon stimulation. **f** Graph represents averaged intensities of Jacob-GFP (*n* = 6). The blue line indicates the trace of fluorescent intensity changes within the nuclear matrix upon field stimulation; the orange line indicates the trace of fluorescent intensity changes at the nuclear lamina. **g**–**i** Field stimulation (18 s@50 Hz) resulted in a simultaneous increase in nuclear calcium, reflecting the pattern of neuronal activity, and a rise in Jacob-CyRFP2 fluorescent intensity. **g** Confocal time-lapse images of hippocampal neuron overexpressing Jacob-CyRFP2 and loaded with Fluo-4 (AM). **f** Averaged normalized intensities of Jacob and Fluo-4 changes upon stimulation. **i** The plot indicates a high correlation between Jacob-CyRFP2 and Fluo-4 (AM) intensities changes upon LTP-like stimuli. Pearson *r* = 0.7997, *p* < 0.0001. **j** Graph represents induction of LTP with TBS in CA1 area of the hippocampus. **k**–**l** TB stimulation induces significant association of pS180-Jacob with CREB as revealed by pJacob-CREB PLA. **l** ^##^*p* < 0.01, Nested *t* test **m** ^##^*p* < 0.01, Nested *t* test, **n–p** TB stimulation results in dissociation of Jacob from LaminB1. **o** ^#^*p* < 0.05, Nested *t* test. **p** ^#^*p* < 0.05, Nested *t* test. **q**–**r** Neurons expressing Jacob at the nuclear lamina and neuronal nuclei characterized by enhanced expression of luciferase as compared to neurons expressing Jacob extranuclear. Schematic representation of the Activity-Responsive Element luciferase reporter (SARE-ArcMin-Luc) where SARE is fused upstream of TATA-containing sequence of the Arc. Panel **q** depicts maximum projection confocal images of cultured hippocampal neurons (DIV16) co-transfected with Jacob and SARE-ArcMin-Luc overnight followed by labeling with anti-Luciferase, anti-MAP2 antibodies and DAPI; scale bar = 10 μm, ***p < 0.001 by *t* test

Phosphorylation of Jacob`s serine residue (S180) encodes in the nucleus activation of synaptic GluN2B-containing NMDAR [38, 54]. Next, we addressed if a brief pulse of synaptic activity would result in association of pS180-Jacob with CREB in the nucleus. As expected, we observed a significant increase in the association of endogenous pS180-Jacob with CREB 5 min after the treatment with 4AP/bicuculline (Fig. 4a–c).

Induction of L-LTP in the CA1 area of the hippocampus results in a rapid increase in [Ca^2+^]_n_ transients [58, 59] crucial for the activation of subsequent transcriptional responses [14, 60, 61]. Stimulation of cultured hippocampal neurons with a 18 s@50 Hz field stimulation protocol produces a stable synaptic potentiation and is highly effective in inducing robust CREB phosphorylation within 30 min in cultured neurons [62]. Therefore, we addressed whether the dissociation of Jacob from the NL and its association with the nuclear matrix are regulated by L-LTP-inducing stimuli. We observed a subnuclear redistribution of Jacob-GFP intensities upon field stimulation (Fig. 4d–f). The increase in GFP fluorescence intensity within the nuclear matrix was observed instantaneously and accompanied by a corresponding detachment of Jacob from the nuclear envelope (Fig. 4d–f). Notably, the pattern of this subnuclear redistribution exactly matched the pattern and time course of stimulation. In stark contrast to basal conditions, however, though Jacob's fluorescence intensity within the nuclear matrix declined following stimulation, as expected, it did not return to baseline and remained elevated throughout the period of recording due to elevated [Ca^2+^]_n_ transients [63].

Next, we applied 50 Hz field stimulation for 18 s and simultaneously recorded [Ca^2+^]_n_ transients and in parallel, the subnuclear distribution of Jacob (Fig. 4g–i; Suppl. Movie 3). As expected, we observed a simultaneous increase in [Ca^2+^]_n_ and enhanced levels of Jacob in the nuclear matrix, suggesting that the pattern of the residing time of the protein reflects the rise in [Ca^2+^]_n_ transients in response to plasticity-inducing stimuli. Taken together, this experiment suggests that Jacob’s subnuclear dynamics initially mimics the “potentiation” pattern, but in response to sustained [Ca^2+^]_n_ elevation, Jacob shows a prolonged association with the nuclear matrix indicating that its nuclear retention persists. Furthermore, we applied theta bursts (TB) to stimulate Schaffer-collateral fibers for induction of LTP in acute hippocampal slices and detected a significant increase in the association of pS180-Jacob with CREB (Fig. 4j–m) that accompanied by the decrease in the association of Jacob with LaminB1 (Fig. 4j, n–p).

Next, to assess the role of Jacob in activity-regulated gene expression, we employed the Synaptic Activity-Responsive Element reporter (SARE-ArcMin-Luc), whose activation is driven in response to Ca^2+^ influx through NMDAR [47]. Since association of Jacob with the nuclear lamina captures the previous history of NMDAR-induced nucleocytoplasmic shuttling [38], we observed enhanced expression of luciferase in neurons expressing Jacob at the nuclear lamina and neuronal nuclei as compared to neurons expressing Jacob extranuclear confirming its role in the regulation of activity-induced genes (Fig. 4q–r).

### Synaptic activity and GluN2B-NMDAR control nucleoplasmic retention of Jacob

Nuclear Ca^2+^ waves are closely coupled to cytosolic Ca^2+^ transients, which are in turn coupled to glutamate release that elicits excitatory postsynaptic currents and Ca^2+^ influx through NMDAR as well as opening of voltage-gated Ca^2+^ channels and backpropagating action potentials [10].

**Fig. 5:**
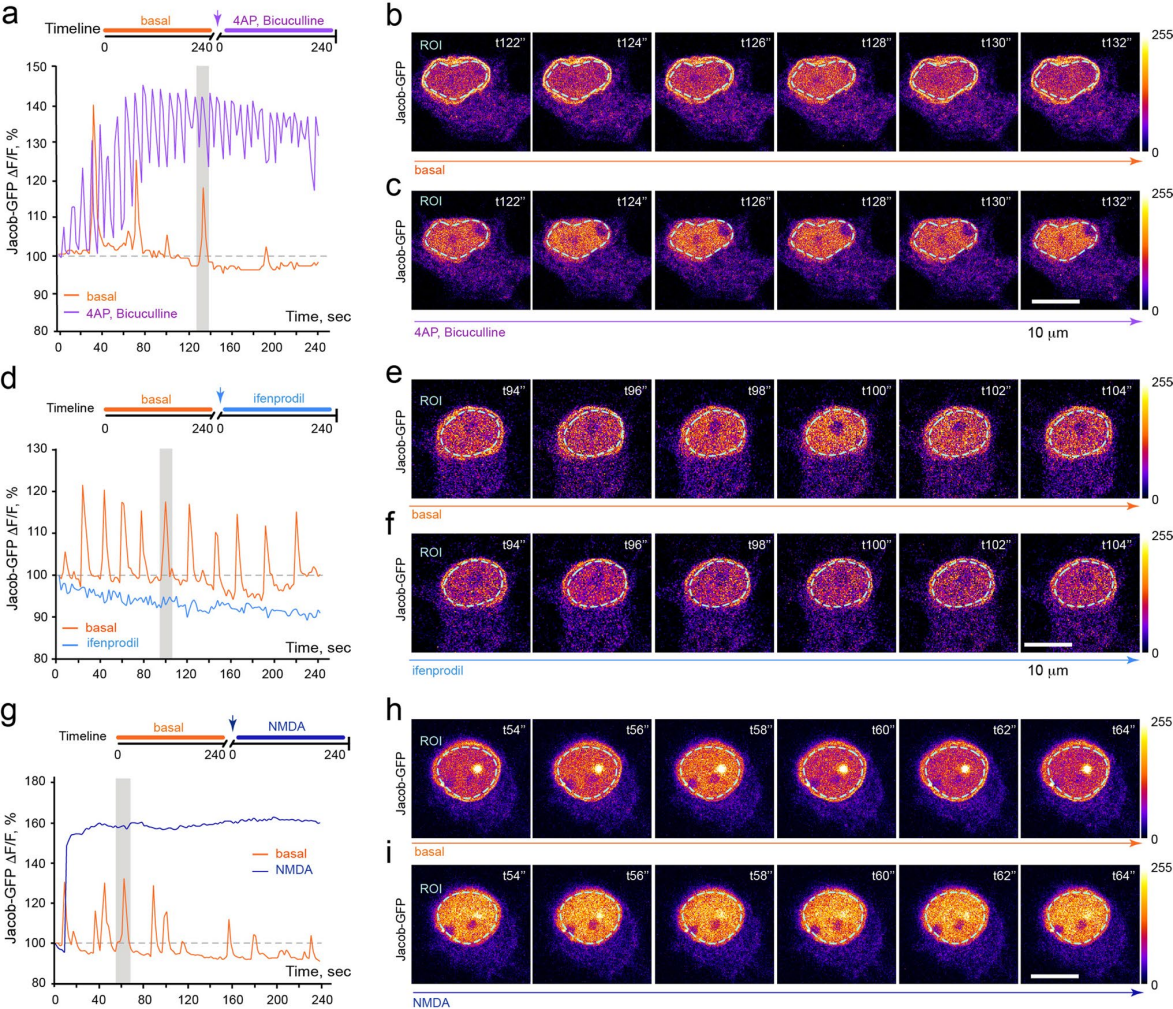
Synaptic activity controls the subnuclear redistribution of Jacob in a GluN2B-dependent manner. **a**–**c** As revealed by time-lapse imaging, enhanced synaptic activity changes the pattern of Jacob’s residing time. **a** Schematic represents the timeline of the experiment. The graph represents changes in fluorescent intensities within indicated ROI over time. Depicted are confocal image frames corresponding to 10 s of recording before (**b**) and after the treatment (**c**) with 4AP/bicuculline (grey bar in **a**). Scale bar = 10 μm. **d–f** Application of ifenprodil abolished the subnuclear redistribution of Jacob during basal glutamatergic transmission. **g–i** Treatment of neurons expressing Jacob-GFP with bath NMDA resulted in plateau-like accumulation of Jacob in the nucleoplasm

Different types of stimulation result in different frequencies and amplitudes of somatic Ca^2+^ transients that encode distinct signals and can be decoded by activation of frequency-sensitive Ca^2+^-sensitive molecules [64, 65]. We next addressed how different patterns of nuclear calcium transients affect Jacob's subnuclear redistribution. Following treatment of neurons with the potassium channel blocker 4-AP (2,5 mM) and the GABA-A receptor antagonist bicuculline (50 μM), a protocol which removes inhibitory tone and results in burst firing of excitatory synapses, we observed robust changes in the association of Jacob with the nuclear matrix (Fig. 5a-c) that were paralleled by enhanced frequency and amplitude of GFP fluorescence in the nucleoplasm (Fig. 5a–c).

Previous work has shown that GluN2B-containing NMDAR activity is crucial for driving Jacob into the nucleus [31, 37]. To learn whether GluN2B-containing NMDAR mediates spontaneous fluctuations of Jacob in the nucleoplasm, we next imaged Jacob-GFP in neurons treated with the GluN2B-NMDAR antagonist ifenprodil. As expected, ifenprodil application abrogated the subnuclear redistribution of Jacob (Fig. 5d–f) whereas bath application of 50 μM NMDA induced a stable increase of Jacob-GFP fluorescence intensity in the nuclear matrix (Fig. 5g–i). Thus, subnuclear Jacob's redistribution reflects the pattern of nuclear calcium transients.

## Discussion

**Fig. 6:**
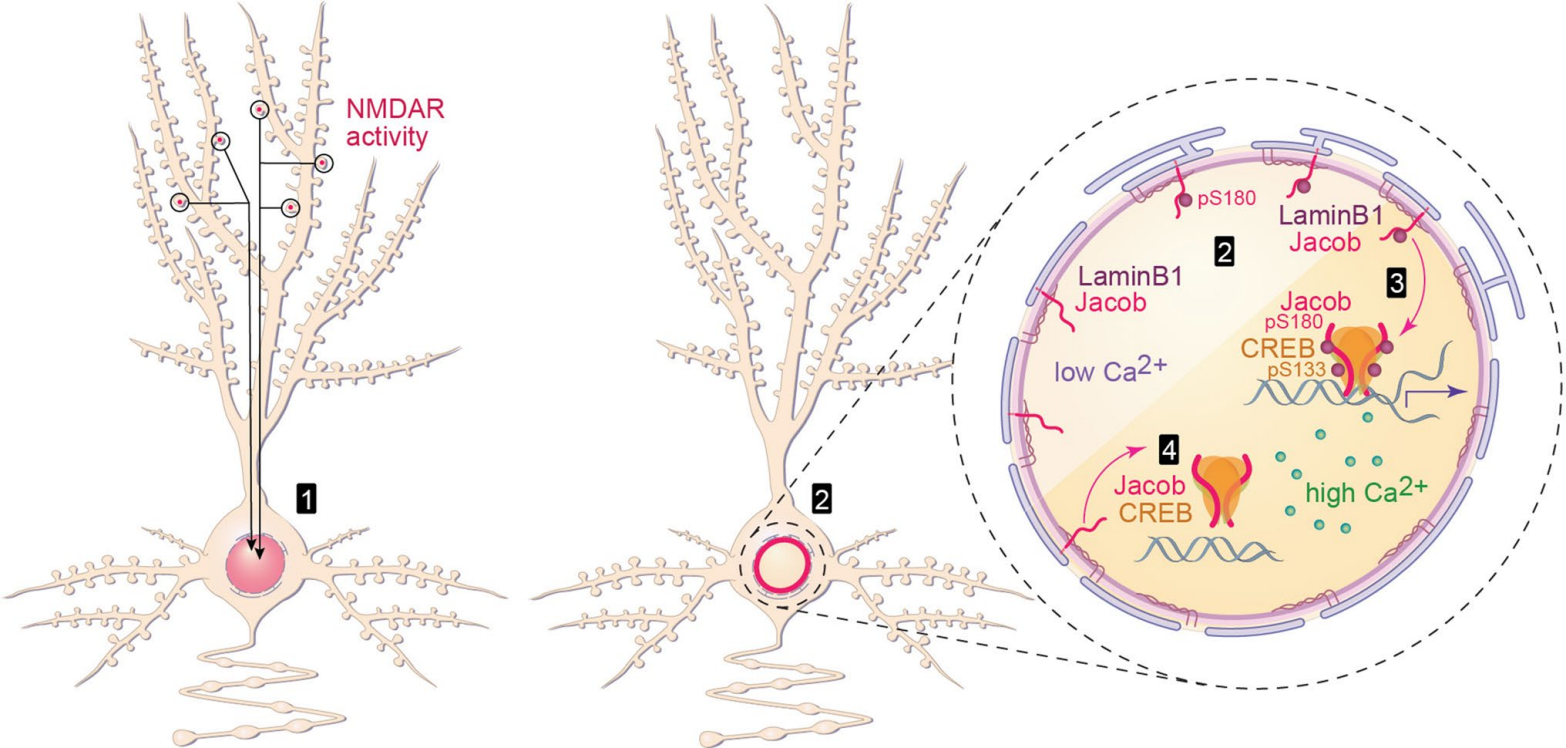
Subnuclear dynamics of the association of Jacob with LaminB1 and CREB are regulated by nuclear [Ca.^2+^]_n_ transients. Synaptic NMDAR activity leads to an active dynein-dependent transport of Jacob from activated synapses to the nucleus (**1**; [31, 37]). In the nucleus, Jacob is present in the nucleoplasm and in association with the nuclear lamina via a direct interaction with LaminB1 [38]. Association of Jacob with LaminB1 captures the history of previous NMDAR activity in the sense that the number of docked molecules reflects previous activation of GluN2B-containing NMDAR. (**2**) Blocking of NMDAR activity with APV and ifenprodil reduced the binding under basal glutamatergic transmission [38]. Concomitantly, an increase in nuclear calcium transients results in liberation of Jacob from the complex with LaminB1 (**3, 4**) which is followed by binding to the transcription factor CREB (**3, 4**)

In this study, we describe how two mechanisms of synapse-to-nucleus communication that play crucial roles in activity-dependent gene expression are eventually integrated in the nucleus (Fig. 6). To the best of our knowledge, we demonstrate for the first time that increased nuclear [Ca^2+^]_n_ transients result in a subnuclear redistribution of a synapto-nuclear protein messenger that has a documented role in transcriptional regulation [30, 31, 37]. Time-lapse imaging of transiently expressed Jacob demonstrated its ability to shuttle between the inner nuclear membrane and the nuclear matrix. Increased nuclear [Ca^2+^]_n_ concentrations liberate Jacob from its association with LaminB1 and thereby promote its binding to CREB (Fig. 6). Intriguingly, this shuttling follows the pattern of nuclear [Ca^2+^]_n_ transients, and docking to the transcription factor is, therefore, likely governed by the pattern of neuronal activity. In this sense, both long-distance signaling pathways are integrated in the nucleus. Of note, although several transcriptional regulators and transcription factors translocate to the nucleus upon increased intracellular Ca^2+^ transients [6, 60, 66–70], for none of them, a dynamic localization at the nuclear rim has been reported with a redistribution to the nuclear matrix upon enhanced nuclear [Ca^2+^]_n_ transients. At present, it is unclear whether this phenomenon has been overlooked or whether this is a unique feature of Jacob.

An important implication of the observed shuttling between the nuclear lamina and the nuclear matrix concerns the integration of different types of signals on Jacob-mediated CREB-dependent gene expression. From previous work, we have learned that the amount of Jacob at the rim bound to LaminB1 reflects the recent history in terms of NMDAR activity [38]. In consequence, neurons with a low number of proteins at the nuclear rim will not be able to respond immediately to the rise in nuclear [Ca^2+^] by binding of Jacob to CREB. On the contrary, neurons with higher levels of synaptic activity will accommodate higher Jacob protein levels at the nuclear periphery. Such an accumulation would boost the impact of previous synapse-to-nucleus communication via protein transport and results in an amplification of the involved signals in case that the signalosome remains stable. Thus, the existence of a stationary but dynamic pool of Jacob at the INM will increase the number of molecules that can readily associate with CREB, which increases the impact of Jacob on CREB-mediated gene expression. Along these lines, the transcription factor CREB is constitutively bound to CRE regulatory sites at some promoters [71] and becomes activated by phosphorylation of its essential residue Ser-133 [6, 72]. We consider it highly likely that Jacob will dock the ERK only to a limited number of targets and the high number of readily available molecules might, thereby, outcompete other binding partners of the bZIP domain like CRTC1. At the same time, ongoing neuronal activity, as reflected in nuclear [Ca^2+^]_n_ transients, has instantaneous impact and thereby overcomes the disadvantage of the time delay that comes along with long-distance transport.

In previous work, we have shown that phosphorylation of serine residue (S180) encodes in the nucleus activation of synaptic GluN2B-containing NMDAR, whereas S180 is not phosphorylated in response to stimulation of extrasynaptic GluN2B-containing NMDAR. The question remains whether Jacob transiently sequestered at the nuclear lamina preserves its phosphorylation status while it is associated with the intermediate filament LaminB1. S180 phosphorylation is crucial for the assembly of a signalosome containing ERK and the intermediate filament α-internexin, which following binding to the basic leucine zipper (bZip) domain of CREB will enhance phosphorylation of S133 and subsequent activation of the transcription factor [31]. Whether the recruitment of phosphorylated Jacob to the nuclear periphery by LaminB1 is beneficial for the preservation of phosphorylation has still to be determined.

In contrast to this scenario, following extrasynaptic NMDAR activation, Jacob is not phosphorylated at S180 and it binds preferentially to the nuclear adaptor protein LIM only 4 (LMO4), a transcriptional co-activator of CREB that hinders de-phosphorylation and transcriptional inactivation of CREB [30]. Jacob interacts with LMO4 in the cytoplasm but also displaces nuclear LMO4 from the CREB complex and thereby renders CREB susceptible to de-phosphorylation. Analogous to the ERK/α-internexin complex, the association with LMO4 strengthens subsequent binding of Jacob to the CREB-phosphatase PP1 and this molecular switch in binding to an adaptor determines sustained transcriptional activation by ERK1/2 or inactivation of CREB by PP1 [30]. It is at present unclear whether these signalosomes will be stable and keep their identity during the residing time of Jacob in the nucleus. Thus, it might be possible that the nuclear Jacob pool switches between binding to ERK and PP1 following activation of synaptic or extrasynaptic NMDAR. In principle, such a switch of adaptor binding is conceivable and vastly different consequences in terms of CREB-dependent gene expression would result from this scenario. One important consequence is that the nuclear Jacob pool would dominate association with CREB because of immediate availability and higher number of molecules that reside in the nucleus as opposed to the low number that undergo long-distance transport.

In any of these scenarios, the question arises how long Jacob will stay in the nucleus and how turnover of the protein is regulated since it has to be expected that this is a major regulatory event. A central observation of the present study was that sustained synaptic activity elicited by LTP-like stimulation results in a persistent shift of Jacob from the nuclear lamina to the nuclear matrix. The underlying molecular mechanisms are unclear but because this shift is very likely involved in the regulation of plasticity-relevant gene expression, future studies should aim to learn more about this process. Moreover, the latter observation points to the possibility that in the nucleus basal synaptic, enhanced synaptic and extrasynaptic NMDAR activity can be discriminated by the subnuclear dynamics of Jacob shuttling. Basal synaptic activity results in dissociation and re-association Jacob with the nuclear lamina (Fig. 6). LTP-like stimulation induces a persistent shift of the Jacob signalosome toward the nuclear matrix, likely containing ERK. Long-lasting activation of extrasynaptic NMDAR like it occurs in disease [73] induces a similar shift of non-phosphorylated Jacob resulting in a long-lasting plateau-like association with CREB and sustained inactivation of the transcription factor (Fig. 6).

In summary, we here report a novel scenario for activity-dependent gene expression that might have significant functional consequences. The tight control of the dynamics of subnuclear localization clearly deserves more attention in future studies.

## Supplementary Information

Below is the link to the electronic supplementary material.

Supplementary file1 (AVI 7128 KB)

Supplementary file2 (AVI 22666 KB)

Supplementary file3 (AVI 17428 KB)

Supplementary file4 Time-lapse imaging of Jacob`s subnuclear redistribution over 10 min. **a** Ten-minute representative trace of Jacob-GFP fluorescence within the nuclear matrix of hippocampal neurons under basal glutamatergic transmission. **b** Depicted are confocal image frames depicting changes in intensities within the nuclear matrix (corresponding to the orange bar in **a**) (JPG 379 KB)

## Data Availability

All data are available upon request. Please contact corresponding authors for data requests.

## Abbreviations

NL: Nuclear lamina
INM: Inner nuclear membrane
NE: Nuclear envelope
LADs: Lamina-associated domains
NMDAR: N-Methyl-d-aspartate receptor
CREB: CAMP response element-binding protein
LMO4: LIM domain only 4
ERK: Extracellular signal-regulated protein kinases
PP1γ: Protein phosphatase 1γ
FRAP: Fluorescence recovery after photobleaching
GFP: Green fluorescent protein
Fluo-4 (AM): Fluo-4 acetoxymethyl ester
CyRFP2: Cyan-excitable red fluorescent protein
PLA: Proximity ligation assay
L-LTP: Late long-term potentiation
GABA-A: γ-Aminobutyric acid
bZip: Basic leucine zipper
CRTC1: CREB-regulated transcription factor
DIV: Days in vitro
HyD: Hybrid detectors
