# Supplementary figures and images for: Integration of nuclear Ca2+ transients and subnuclear protein shuttling provides a novel mechanism for the regulation of CREB-dependent gene expression
Source: Cell Mol Life Sci. 2023 Jul 25;80(8):228. doi: 10.1007/s00018-023-04876-8 (PMC10368568; doi:10.1007/s00018-023-04876-8)

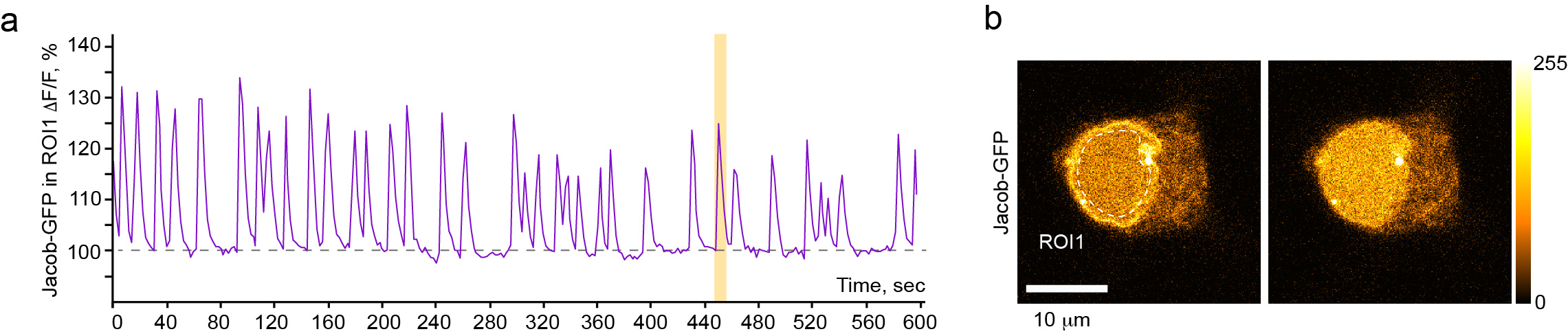

Supplement: Supplementary file 4 — Supplementary file4 Time-lapse imaging of Jacob`s subnuclear redistribution over 10 min. a Ten-minute representative trace of Jacob-GFP fluorescence within the nuclear matrix of hippocampal neurons under basal glutamatergic transmission. b Depicted are confocal image frames depicting changes in intensities within the nuclear matrix (corresponding to the orange bar in a) (JPG 379 KB) [file 18_2023_4876_MOESM4_ESM.jpg]
